# Improving Lead Phytoremediation Using Endophytic Bacteria Isolated from the Pioneer Plant *Ageratina adenophora* (*Spreng.*) from a Mining Area

**DOI:** 10.3390/toxics12040291

**Published:** 2024-04-16

**Authors:** Qiqian Li, Siyu Yao, Hua Wen, Wenqi Li, Ling Jin, Xiuxiang Huang

**Affiliations:** 1 College of Chemical and Biological Engineering, Hechi University, Hechi 546300, China; liqiqian@hcnu.edu.cn (Q.L.);; 2 Guangxi Key Laboratory of Sericulture Ecology and Applied Intelligent Technology, School of Chemistry and Bioengineering, Hechi University, Hechi 546300, China; 3 Guangxi Collaborative Innovation Center of Modern Sericulture and Silk, School of Chemistry and Bioengineering, Hechi University, Hechi 546300, China; 4 Department of Civil and Environmental Engineering, The Hong Kong Polytechnic University, Hung Hom, Kowloon 999077, Hong Kong; yaosy19@lzu.edu.cn (S.Y.);; 5 Department of Health Technology and Informatics, The Hong Kong Polytechnic University, Hung Hom, Kowloon 999077, Hong Kong

**Keywords:** heavy metals, phytoremediation, plant growth-promoting bacteria, *Sphingomonas* sp.

## Abstract

This study aimed to isolate and characterise endophytic bacteria from the pioneer plant *Ageratina adenophora* in a mining area. Seven strains of metal-resistant endophytic bacteria that belong to five genera were isolated from the roots of *A. adenophora*. These strains exhibited various plant growth-promoting (PGP) capabilities. *Sphingomonas* sp. ZYG-4, which exhibited the ability to secrete indoleacetic acid (IAA; 53.2 ± 8.3 mg·L^−1^), solubilize insoluble inorganic phosphates (Phosphate solubilization; 11.2 ± 2.9 mg·L^−1^), and regulate root ethylene levels (1-aminocyclopropane-1-carboxylic acid deaminase activity; 2.87 ± 0.19 µM α-KB·mg^−1^·h^−1^), had the highest PGP potential. Therefore, *Sphingomonas* sp. ZYG-4 was used in a pot experiment to study its effect on the biomass and Pb uptake of both host (*Ageratina adenophora*) and non-host (*Dysphania ambrosioides*) plants. Compared to the uninoculated control, *Sphingomonas* sp. ZYG-4 inoculation increased the biomass of shoots and roots by 59.4% and 144.4% for *A. adenophora* and by 56.2% and 57.1% for *D. ambrosioides*, respectively. In addition, *Sphingomonas* sp. ZYG-4 inoculation enhanced Pb accumulation in the shoot and root by 268.9% and 1187.3% for *A. adenophora*, and by 163.1% and 343.8% for *D. ambrosioides*, respectively, compared to plants without bacterial inoculation. Our research indicates that endophytic bacteria are promising candidates for enhancing plant growth and facilitating microbe-assisted phytoremediation in heavy metal-contaminated soil.

## 1. Introduction

Heavy metal (HM) contamination and soil degradation caused by rapid industrialisation and urbanisation has become a critical environmental issue. Extensive mining and smelting activities and related dumping are major causes of metal contamination in soil and have severe implications on the natural ecosystem services, ultimately posing a significant threat to human health via the food chain [1]. Pb is a highly toxic and carcinogenic heavy metal that is non-biodegradable and poses a significant risk to human health [2]. Exposure to Pb can result in neurological and organ damage, leading to adverse health outcomes such as gastrointestinal impairments, childhood cognitive deficits, and Alzheimer’s disease [3]. To maintain ecological security, it is necessary to undertake measures for the remediation of Pb-contaminated soil, as well as to implement strategies aimed at preventing the harmful effects associated with biomagnification in the food chain.

Phytoremediation has emerged as a promising technique for the remediation of Pb-contaminated soil owing to its economic and environmental friendliness. [4]. Screening for efficient metal-hyperaccumulating plants with a high heavy metal resistance and transfer coefficient is essential in applications of phytoremediation [5]. To date, over 20 plant species, including *Microstegium ciliatum*, *Polygala umbonate*, *Spermacoce mauritiana*, *Sedum alfredii*, *Alyssum wulfenianum*, *Rhododendron annae*, *Llex plyneura*, and others, have been identified as Pb-hyperaccumulating plants and researchers continue to search for new hyperaccumulators [6,7,8,9]. However, most heavy metal hyperaccumulators suffer from drawbacks such as low biomass and slow growth, which can compromise the effectiveness of phytoremediation [10]. Endophytic bacteria colonize the interior of plant tissues and form long-term mutualism with their hosts [11]. They show a high metabolic capacity and metabolic specificity as critical components of the plant microbiome, playing an important role in enhancing plant adaptation to the environment, improving plant growth, and collaborating with host plants to tolerate heavy metal stress [12]. As a novel phytoremediation strategy, endophytic bacteria assisted-phytoremediation has shown incredible potential in the remediation of heavy metal-contaminated soils.

Plant-growth promoting (PGP) bacteria, a type of beneficial endophytic bacteria, can enhance the plants’ resistance to heavy metals by solubilizing phosphorus, nitrogen fixation, secreting growth regulators, synthesizing siderophores, and improving plant tolerance to heavy metal stress [13]. PGP bacteria can release siderophores, organic acids that may chelate free heavy metal ions, making them less likely to be transported into plant cells and reducing the toxic effects of heavy metals on plants by altering their bioavailability [14]. PGP bacteria can also reduce the ethylene content in plant roots under metal stress by secreting 1-aminocyclopropane-1-carboxylic acid (ACC) deaminase, which catalyses the hydrolysis of the ethylene precursor ACC into α-ketobutyrate and ammonia, thereby increasing plant resistance to metal stress [15]. In addition, PGP bacteria can provide plants with nitrogen and phosphorus through phosphate solubilization and nitrogen fixation, or they can synthesise plant hormones such as indoleacetic acid (IAA) and cytokinin to promote plant growth and increase plant biomass, thereby indirectly mitigating the effects of heavy metal stress [16]. PGP bacteria evolved from host-plant rhizosphere microbiomes under long-term heavy metal stress and formed a stable and intimate mutualistic symbiosis with host plants. This mutually beneficial relationship is not specific, and certain endophytes may colonize and promote the growth of a variety of non-host plant tissues [17]. Exploring the effect of combined remediation by endophytic bacteria and non-host plants will significantly expand the scope and value of phytoremediation applications for heavy metal contamination.

Endophytic bacteria with PGP characteristics are increasingly being studied for their role in phytoremediation. Numerous PGP bacteria have been reported to promote plant development and heavy metal tolerance [16,18,19]. However, only a few studies have explored their effects on plant growth and Pb accumulation. Furthermore, the impact of plant PGP bacteria on the growth of non-host plants and their phytoremediation potential in metal-contaminated soils remains poorly understood. *A. adenophora* is a typical pioneer species in mining areas, with rapid growth, high resistance, and high Pb uptake capacities [20]. Therefore, *A. adenophora* is a promising plant for the phytoremediation of Pb-contaminated areas. However, current research on the remediation of heavy metal pollution by *A. adenophora* is limited to the analysis of heavy metal enrichment and transport effects as well as physiological and biochemical indices, and little research has been conducted on the endophytic bacteria-assisted *A. adenophora* plants’ remediation of heavy metal-polluted soil. The objectives of this study were to (1) isolate Pb-resistant endophytic bacteria from *A. adenophora* root tissues harvested from a Pb mine tailing, (2) characterise the PGP traits of isolated endophytic bacteria, and (3) investigate the improvement in lead phytoremediation in both the host plants (*A. adenophora*) and non-host plants (*D. ambrosioides*) by the selected strain with the best PGP potential.

## 2. Materials and Methods

### 2.1. Plant Materials

The *A. adenophora* plants used for screening metal-resistant endophytes were collected from a Pb mine tailing located in the northwest of Nandan County (25°13′ N, 107°25′ E), Hechi city, China. Geochemical conditions of the sampling site were as follows (Table S1): pH, 7.5; total organic carbon, 75.1 mg·kg^−1^; total N, 22.4 mg·kg^−1^; total P, 1.05 mg·kg^−1^; Pb, 1242 mg·kg^−1^; Cd, 376 mg·kg^−1^; Cu, 345 mg·kg^−1^; Zn, 730 mg·kg^−1^; As, 772 mg·kg^−1^. Ten plants were collected using a 75% (*v*/*v*) ethanol-sterilized shovel and were transported to the laboratory in dry ice, kept at 4 °C, then processed within 24 h. Mature seeds of *A. adenophora* and *D. ambrosioides* were collected from the same location. They were soaked in distilled water for 12 h to soften the seed coat and subsequently sown in trays containing sterilized sand about 2 inches thick for germination. After two weeks of incubation in a greenhouse at 22–25 °C, seedlings of uniform morphology were transferred for pot experiments.

### 2.2. Isolation and Identification of Metal-Resistant Endophytes

Metal-resistant endophytes were isolated from *A. adenophora* roots using a previously described modified method [14]. In brief, the collected *A. adenophora* plant samples underwent thorough washing with tap water, followed by three rinses with deionized water. Healthy root tissue was sterilized by incubating in 75% (*v*/*v*) ethanol and 3% sodium hypochlorite solution for 2 min each. Subsequently, the root tissue underwent three rinses in sterile distilled water and was then dried on sterilized filter paper. The third rinse water was plated on LB medium (1% Peptone, 0.5% Yeast Extract, 1% NaCl, and 1.5% Agar; pH 7.2) to assess surface disinfection effectiveness. Surface-sterilised root tissues were then transferred to a mortar with 5 mL of sterile phosphate-buffered saline (pH 7.4) and ground to mixed samples. The extracted liquid was diluted and plated onto LB medium supplemented with 100 mg·L^−1^ Pb, and subsequently incubated at 30 °C for a duration of 3 days.

To identify each isolate, genomic DNA extraction was carried out using the Sangon Bacterial DNA Kit (Sangon Biotech, Shanghai, China) and PCR amplification targeted the 16S rRNA gene using the universal primers 27F (5′-AGAGTTTGATCMTGGCTCAG-3′) and 1492R (5′-TACGGHTACCTTGTTACGACTT-3′) [21]. The details of PCR experimental system and thermal cycling protocol were elucidated in the Supplementary Methods S1. Sequences of the amplicons were obtained from Sangon Biotech (Shanghai, China), and these sequences were matched to GenBank nucleotide sequences according to the BLASTn program [22]. Phylogenetic analysis was based on MEGA6 software (Version 6.0) with the neighbour-joining method [23].

### 2.3. Pb Tolerance of the Isolated Strains

Pb tolerance of the isolated strains were determined as follows: LB agar plates were inoculated with all isolates, and Pb concentrations ranging from 100 to 1000 mg·L^−1^ were added individually. Cultures were incubated at 30 °C for 5 days, with the minimum inhibitory concentration (MIC) defined as the lowest concentration of lead (Pb) at which bacterial growth ceased to be observed [24].

### 2.4. Characterization of PGP Traits of Endophytes

The method developed by Fan et al. [25] was utilized to quantify the production of indole acetic acid (IAA). Briefly, the endophytes were inoculated into a sucrose minimal salts (SMS) medium supplemented with 500 mg·L^−1^ of tryptophan and then incubated at 30 °C for a duration of 4 days. Following the incubation period, 1 mL aliquot of the cell suspension was transferred to a tube and vigorously mixed with 2 mL of Salkowski’s reagent, which consists of 150 mL of concentrated H_2_SO_4_, 250 mL of distilled water, and 7.5 mL of 0.5 mol FeCl_3_·6H_2_O. This mixture was allowed to stand at room temperature for 20 min. The concentration of IAA in the culture medium was determined by spectrophotometry at 600 nm.

To detect ACC deaminase activity, the endophytic strains were inoculated into tubes containing 10 mL of Dworkin and Foster (DF) medium, wherein (NH_4_)_2_SO_4_ was replaced with 3 mmol ACC as the nitrogen source [26]. Subsequent to incubation at 30 °C for 72 h, the bacterial cells were harvested via centrifugation at 7878× *g* for 10 min at 4 °C. The ACC deaminase activity of these cells was determined by quantifying the amount of α-ketobutyrate generated through the enzymatic hydrolysis of ACC, following the procedure by Sheng et al. [27].

Modified Pikovskaya’s medium enriched with tricalcium phosphate, was employed to assess the phosphate solubilization ability of the endophytes [28]. All isolates were incubated at 30 °C for 3 days and the amount of solubilized phosphate in the culture supernatant was measured spectrophotometrically at 700 nm using the Mo blue method [29].

The secretion of siderophores by the endophytic strains was evaluated using the method developed by Schwyn and Neilands, which utilizes blue agar plates containing the dye chrome azurol S (CAS) [30]. After spotting the endophytes on CAS agar plates, they were then incubated at 30 °C for a duration of 6 days. A positive indication of siderophore production was observed through the presence of orange halos surrounding colonies on the blue agar plates.

The details of PGP traits’ analytical methods were given in the Supplementary Methods S2.

### 2.5. Pot Experiment

Pot experiment soil was sampled from an agricultural field in Hechi City, China (24°29′ N, 108°39′ E). The physicochemical characteristics of the soil were recorded as follows: pH, 7.5; total organic carbon, 15.5 g·kg^−1^; total N, 0.39 g·kg^−1^; total K, 2.33 g·kg^−1^; available P, 6.9 mg·kg^−1^; Pb, 1.18 mg·kg^−1^. After air drying at room temperature and sieving, the soil was treated with PbCl_2_ solutions to achieve final concentrations of Pb, 200 mg·kg^−1^ or 1000 mg·kg^−1^. The amended soil was placed in a greenhouse and stabilised for 2 weeks. The morphologically uniform seedlings (five seedlings/pot) of *A. adenophora* or *D. ambrosioides* were planted in plastic pots containing 2 kg of natural soil or amended soil. For endophytes inoculation [18], PGP bacteria ZYG-4 was acquired from LB liquid culture during its exponential growth phase. Following centrifugation to clear the medium, the ZYG-4 inoculum was prepared by suspending the cells in sterilised saline solution (0.85%) to achieve an inoculum density of 10^7^ cfu·ml^−1^. Five treatment groups with three replicates each were conducted as follows: *A. adenophora* or *D. ambrosioides* were planted in natural soil (CK); 200 mg·kg^−1^ Pb-contaminated soil (Pb200); 200 mg·kg^−1^ Pb-contaminated soil inoculated with PGP bacteria ZYG-4 (Pb200Z); 1000 mg·kg^−1^ Pb-contaminated soil (Pb1000); 1000 mg·kg^−1^ Pb-contaminated soil inoculated with PGP bacteria ZYG-4 (Pb1000Z). All treatment groups were cultured in a greenhouse with a day/night temperature of 25 °C/18 °C and soil humidity was maintained around 60%. For treatment groups inoculated with PGP bacteria, the endophytes were gently inoculated into the rhizosphere soil through weekly drenching with 50 mL of ZYG-4 inoculum.

### 2.6. Analysis of Plant Biomass and Pb Uptake

After an experimental period of 60 days, the harvested plants were carefully washed in ultrapure water and divided into shoot and root parts. The samples were individually dried in an oven at 80 °C for a duration of 24 h, then the dry biomass of each sample was measured and recorded. The plant tissues (0.2 g) were crushed and digested in a microwave digester (Milestone ETHOS PLUS, Sorisole, Italy) with HClO_4_ and HNO_3_ (1:4, *v*/*v*) at 180 °C for 30 min. Pb content of the solution was quantified by atomic absorption spectrometry (ZEEnit 770P, Analytik, Jena, Germany) after filtration.

### 2.7. Statistical Analysis

Statistical analysis was carried out utilizing SPSS 18.0 (SPSS Inc., Chicago, IL, USA) for Windows. One-way analysis of variance (ANOVA) was conducted to determine the effect of the introduced strains on plant growth parameters and Pb content in plant tissues. Significance was attributed to differences between samples with a *p*-value < 0.05.

## 3. Results

### 3.1. Isolation of Pb and Cd Resistant Endophytic Bacterium

**Figure 1:**
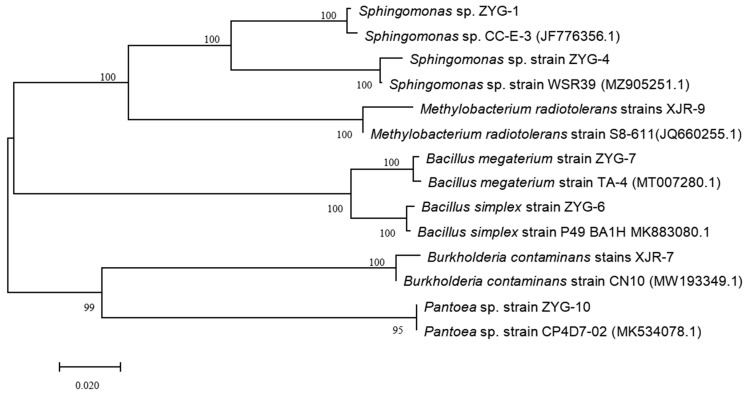
Phylogenetic tree of metal-tolerant endophytic bacteria associated with *A. adenophora* based on the 16S rDNA sequences.

Fifteen strains were isolated from the surface-sterilized roots of the host plant *A. adenophora* after enrichment, isolation, and purification. Seven of the screened endophytes were able to grow on the screening medium with 50 mg·L^−1^ Pb. They were named ZYG-1, ZYG-4, ZYG-6, ZYG-7, ZYG-10, XJR-7, and XJR-9, respectively. According to the sequencing results of the 16S rRNA gene, these bacterial isolates were categorized into five genera belonging to the phyla *Proteobacteria* and *Firmicutes*. Figure 1 shows the phylogenetic relationships of these metal-tolerant endophytic bacteria. The ZYG-1 and ZYG-4 isolates were closest to *Sphingomonas* sp.; the ZYG-6 and ZYG-7 isolates clustered in the same branch with *Bacillus* sp. strains; and the XJR-7, XJR-9, and ZYG-10 isolates belonged to the genera *Burkholderia contaminans*, *Methylobacterium radiotolerans*, and *Pantoea* sp., respectively.

**Figure 2:**
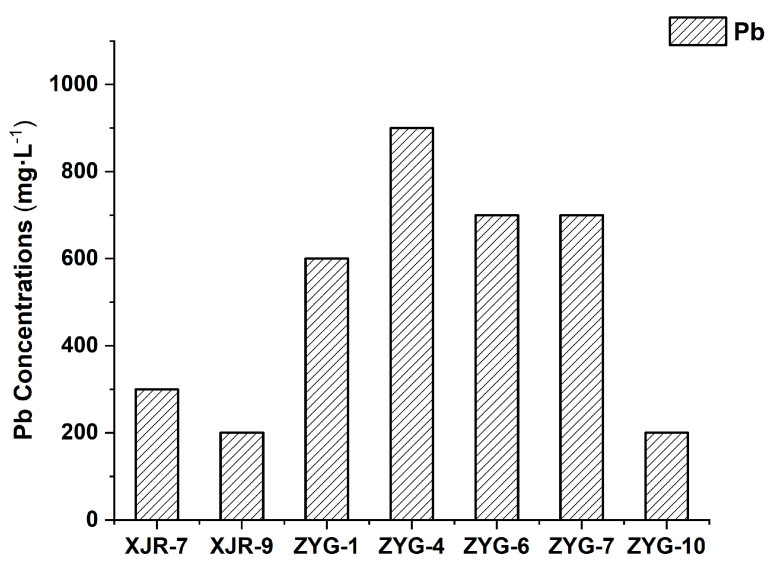
The minimum inhibitory concentration (MIC) values for Pb of endophytic bacteria isolated from *A. adenophora*.

The minimum inhibitory concentration (MIC) values of these endophytic bacteria isolated from *A. adenophora* were determined in response to Pb exposure (Figure 2). All the selected endophytic strains exhibited a high level of tolerance to lead ions, with MIC values ranging from 200 to 900 mg·L^−1^. Among these isolates, ZYG-4, belonging to the genus *Sphingomonas*, showed the highest tolerance to Pb, tolerating up to 900 mg·L^−1^ of lead.

### 3.2. PGP Characteristics of the Endophytic Bacteria

The PGP traits of all the isolates were evaluated and are presented in Table 1. Among the seven isolates, all could produce IAA within the range of 2.5–53.2 mg·L^−1^, with notable production levels observed for XJR-7 and ZYG-4, exceeding 35 mg·L^−1^. In ACCD assay, the isolate ZYG-4 which produced 2.87 ± 0.19 μmol α-ketobutyrate·mg protein^−1^·h^−1^ exhibited the highest ACC deaminase activity. In addition, the isolates ZYG-7 and ZYG-10 also degraded ACC well, with ACC deaminase activities of 1.81 and 1.43 μmol α-ketobutyrate·mg protein ^−1^·h^−1^, respectively. All isolated endophytic strains solubilized mineral phosphate at different levels. Among these, ZYG-1 solubilized the most phosphate, up to 20.8 ± 1.2 mg·L^−1^. Followed by ZYG-4, which had a phosphate dissolving capacity of 11.2 ± 2.9 mg·L^−1^. Screening for siderophore production on CAS agar plates showed that 57% of the total isolates produced siderophores.

**Figure 3:**
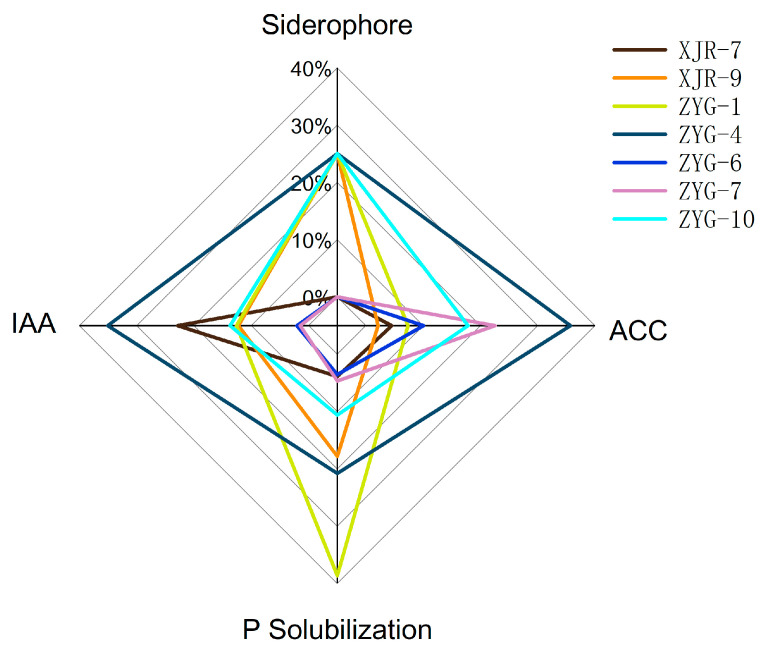
Radar chart of the plant-growth promoting activities with isolated endophytes.

To select the most optimal candidate from the isolated endophytes, we evaluated PGP traits using radar plots of the above four indexes’ percentage data (Figure 3). The results suggest that ZYG-4 of the genus *Sphingomonas* has the best growth-promoting potential, and thus we chose it as the inoculum for the next potting experiment.

### 3.3. Effect of PGP Bacteria ZYG-4 on the Biomass in Plants

**Figure 4:**
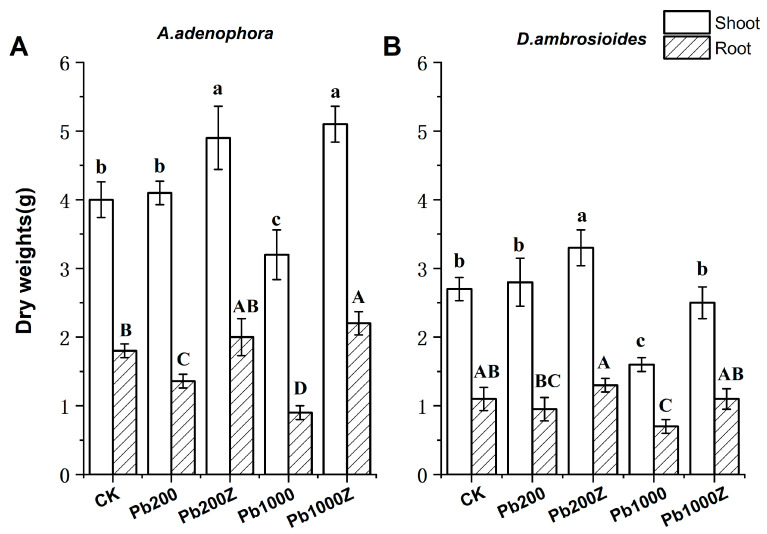
Effects of the endophyte ZYG-4 inoculation on dry weights of plants. (**A**) Host plant (*A. adenophora*); (**B**) Non-host plant (*D. ambrosioides*). CK, natural soil; Pb200, artificial soil with lead concentration of 200 mg·kg^−1^; Pb200Z, artificial soil inoculated with ZYG-4 at a lead concentration of 200 mg·kg^−1^; Pb1000, artificial soil with lead concentration of 1000 mg·kg^−1^; Pb1000Z, artificial soil inoculated with ZYG-4 at a lead concentration of 1000 mg·kg^−1^. The Duncan test indicates significant differences (*p* < 0.05) in dry weights, as denoted by both capital and lowercase letters. Capitals represent variations in root dry weights, while lowercases denote distinctions in shoot dry weights. The vertical bars represent the standard deviation (*n* = 3).

Inoculation with live cells of *Sphingomonas* sp. ZYG-4 enhanced plant growth significantly. *A. adenophora* inoculated with ZYG-4 had a 19.5% increase in shoots dry weight compared to the control plants without PGP bacteria treatment in soil contaminated with 200 mg·kg^−1^ of lead. The trend of increasing shoots biomass caused by inoculation of ZYG-4 was more prominent in high contaminated soil. ZYG-4 inoculation increased the shoot biomass of *A. adenophora* by about 59.4% in soil with 1000 mg·kg^−1^ of lead. Inoculation with PGP bacteria ZYG-4 also enhanced the roots biomass of *A. adenophora*. The root dry biomass of *A. adenophora* treated with ZYG-4 increased by 42.8% and 144.4%, respectively, in soil contaminated with 200 and 1000 mg·kg^−1^ of Pb, compared to the control plants (Figure 4A). Figure 4B shows that ZYG-4 inoculation also increased the biomass of non-host plants (*D. ambrosioides*). Inoculation increased the shoots biomass of *D. ambrosioides* by 17.8 and 56.2%, and the roots biomass by 30 and 57.1%, respectively, in soil contaminated with 200 and 1000 mg·kg^−1^ of Pb.

### 3.4. Effect of PGP Bacteria ZYG-4 on Phytoremediation of Pb

**Figure 5:**
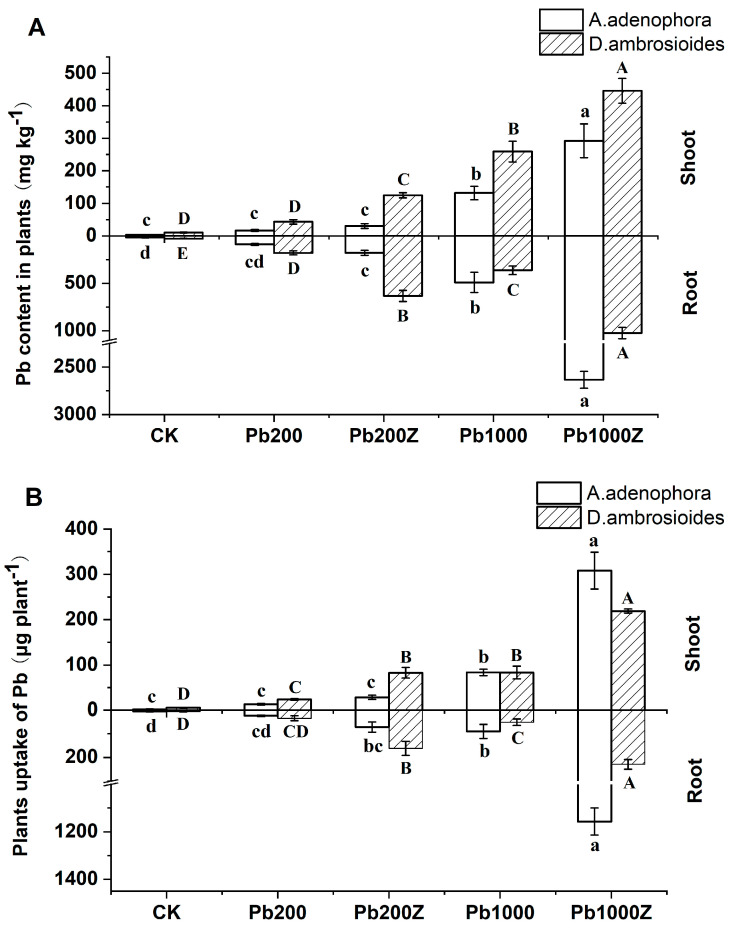
Effects of the endophyte ZYG-4 inoculation on Pb content (**A**) and uptake (**B**) in host (*A. adenophora*) and non-host plants (*D. ambrosioides*). CK, natural soil; Pb200, artificial soil with lead concentration of 200 mg·kg^−1^; Pb200Z, artificial soil inoculated with ZYG-4 at a lead concentration of 200 mg·kg^−1^; Pb1000, artificial soil with lead concentration of 1000 mg·kg^−1^; Pb1000Z, artificial soil inoculated with ZYG-4 at a lead concentration of 1000 mg·kg^−1^. The different letters indicate significant differences at the 0.05 level (one-way ANOVA followed by the Duncan’s test). Capitals represent variations in *A. adenophora*, while lowercases denote distinctions in *D. ambrosioides*. The vertical bars represent the standard deviation (n = 3).

Inoculation with endophytic strain ZYG-4 affected the Pb concentration in the host plants (*A. adenophora*) and non-host plants (*D. ambrosioides*) to varying degrees (Figure 5). In two types of lead-contaminated soils, ZYG-4 increased the Pb concentration in the roots of the *A. adenophora* to 178.6 and 2633.2 mg·kg^−1^, respectively, 102.2% and 436.3% higher than the control group without bacteria inoculation. The concentration of lead (Pb) in the shoots of the *A. adenophora* increased by 82.5% and 121.7% after inoculation with ZYG-4 in 200 and 1000 mg·kg^−1^ Pb-contaminated soil, respectively. Simultaneously, the inoculation treatment resulted in a significant increase in the Pb concentration in *D. ambrosioides*. In 1000 mg·kg^−1^ Pb-contaminated soil, the Pb concentration in its shoots and roots increased by 72.0% and 182.3%, respectively, with the inoculation of ZYG-4 (Figure 5A).

Although ZYG-4 showed different effects on the Pb concentration in different parts of plants, the inoculation treatment increased the biomass of the plant, and thus the total amount of Pb enrichment in the plant was improved to different degrees compared to the control. In Pb200Z treatment, ZYG-4 increased the total amounts of Pb enrichment in the roots with each plant of *A. adenophora* (196.7%) and *D. ambrosioides* (367.2%) compared with the control group and increased the total Pb accumulation in the shoots (116.1% and 274.2%), respectively. Furthermore, the total accumulation of Pb in plant tissues increased progressively with the soil lead concentration. In Pb1000Z treatment, ZYG-4 inoculation enriched 1156.7 and 229.1 μg·plant^−1^ of Pb in the roots of *A. adenophora* and *D. ambrosioides*, respectively, 1187.3 and 343.8% higher than the control (*p* < 0.05). Concurrently, ZYG-4 inoculation also enriched 308.1 and 218.9 μg·plant^−1^ of Pb in the shoots of *A. adenophora* and *D. ambrosioides*, respectively, 268.9% and 163.1% more than the control without bacteria inoculation (Figure 5B).

## 4. Discussion

Endophytic bacteria exhibiting PGP traits can facilitate phytoremediation by enhancing plant growth, reducing heavy metal stress, and increasing heavy metal accumulation. Thus, screening and isolating promising PGP bacteria is essential to improve the efficiency of phytoremediation. In the present study, seven metal-resistant endophytic bacteria belonging to five distinct genera were isolated from the roots of *A. adenophora* in a Pb mine. Although all seven isolates were previously reported as PGP bacteria and used for the phytoremediation of heavy meal-contaminated soils [31,32,33,34], to our knowledge, this is the first report and reference to PGP bacteria *Methylobacterium radiotolerans* involved in lead phytoremediation. An evaluation of the PGP characteristics of the endophytes that we isolated revealed that the majority of them had the potential to promote plant growth to a certain degree. Among them, isolate *Sphingomonas* sp. ZYG-4 exhibited the highest Pb tolerance efficacy and the strongest growth-promoting potential and was chosen as the valuable endophytic bacterium for further study. *Sphingomonas* are widespread in the rhizosphere and endosphere of mining plants. Previous studies have demonstrated the significance of the *Sphingomonas* genus in enhancing plant tolerance to abiotic stress, making it a promising candidate for microbe-assisted phytoremediation approaches [35]. *Sphingomonas* sp. demonstrate potential for heavy metal phytoremediation through various mechanisms during microbe–plant symbiosis. These mechanisms include the enhancement of plants’ heavy metals tolerance, regulation of endogenous phytohormones, secretion of bacterial siderophores, suppression of ethylene production in plants, and improved rhizosphere nutrient bioavailability under stress conditions. Our experimental results shows that the strain *Sphingomonas* sp. ZYG-4 produces indoleacetic acid (IAA; 53.2 ± 8.3 mg·L^−1^) and has the ability to solubilize insoluble inorganic phosphates (Phosphate solubilization; 11.2 ± 2.9 mg·L^−1^) and regulate root ethylene levels (ACC deaminase activity; 2.87 ± 0.19 µM α-KB·mg^−1^·h^−1^). In comparison to the recent report on *A. adenophora* endophytes, the strain *Sphingomonas* sp. ZYG-4 had better PGP characteristics [36]. The probable explanation for this is that, in the current study, the *A. adenophora* plants used for endophytes isolation were sampled from Pb mine tailing which was an extreme oligotrophic ecosystem, and it is likely that these endophytes were generated with a stronger plant-growth promoting capability to adapt to the environment during long-term natural selection.

Inoculating plants with PGP bacteria can affect their uptake and accumulation of heavy metals. Wang et al. [37] found that inoculating *Brassica juncea* with *Sphingomonas* SaMR12 significantly enhanced its Cd uptake. Praburaman et al. [38] reported that *Herbaspirillum* sp. GW103 inoculation enhanced Pb and Zn accumulation in *Zea mays* L. shoots and roots by 27% and 84%, respectively. In the present study, we inoculated plants with *Sphingomonas* sp. ZYG-4 and observed a significant increase in their Pb accumulation (Figure 5). One possible explanation for this result is that inoculation with PGP bacteria increased the biomass and root system of the plants, which may have enhanced Pb accumulation and nutrient absorption. Alternatively, PGP bacteria inoculation may modify Pb solubilization and alter the rhizosphere’s physical and chemical properties, ultimately enhancing metal uptake and Pb accumulation. Most PGP bacteria produce siderophores, which exhibit a high affinity for divalent metal ions, thereby influencing the bioavailability of heavy metals in soils. For instance, it was reported that the PGP bacteria *B.pumilus* E2S2 has been observed to enhance the extraction of Cd from soil, which can potentially be attributed to its capacity for siderophore production in both catechol and hydroxamate types [39]. Our pot experiment results indicate that *Sphingomonas* sp. ZYG-4 can enhance plant Pb accumulation, providing a robust candidate for microbe-assisted phytoremediation strategies. Although PGP bacteria have been widely reported to promote plant development and enhance plant remediation efficiency [40,41,42,43], there is limited knowledge regarding their efficacy in enhancing the growth of non-host plants, especially under heavy metal stress conditions [44]. In this study, we isolated *Sphingomonas* sp. ZYG-4 from the host plant *A. adenophora*. This plant is a pioneer species in mining areas with promising phytoremediation potential. However, it is also an invasive plant species that threatens natural ecosystems [45]. Therefore, using the PGP bacteria *Sphingomonas* sp. ZYG-4 to assist the host plant *A. adenophora* in phytoremediation may cause new ecological risks, which makes research on enhancing phytoremediation of non-host plants more important. Ma et al. [46] found that *Pseudomonas* sp. A3R3, isolated from the root tissues of Ni hyperaccumulator *A. serpyllifolium*, were able to enhance root development and promote growth in the non-host plant *B. juncea*. Plociniczak et al. [47] also discovered that *Proteus vulgaris* H7 and *Pseudomonas* sp. H15, obtained from *Silene vulgaris*, exhibited transient colonization of white mustard roots and facilitated the phytoextraction of Cd and Zn. Nevertheless, not all PGP bacteria have been shown to have growth promotion effects on both host and non-host plant species. Certain PGP bacteria may be host-specific and affect the growth of non-host plants adversely or slightly [37,48]. Therefore, it is essential to investigate the screened endophytes that exhibit plant growth-promoting effects on non-host plants to facilitate the development of novel and effective soil remediation agents. Previous studies reported that *D. ambrosioides* is a lead hyperaccumulator, but its phytoremediation efficiency is limited by its low biomass and shallow root system [49,50]. In the present study, inoculation with the isolated *Sphingomonas* sp. ZYG-4 significantly enhanced the growth and biomass of *D. ambrosioides* under stress, especially with high heavy metal concentrations (Figure 4B). PGP bacteria addition significantly increased shoot and root biomass by a maximum of 56.2% and 57.1%, respectively. Additionally, inoculation with *Sphingomonas* sp. ZYG-4 led to a substantial enhancement of Pb accumulation in the shoot and root by 163.1% and 343.8%, respectively, compared to plants without bacterial inoculation. Our study first examined the effects of the isolated *Sphingomonas* sp. ZYG-4 on non-host plants *D. ambrosioides* and found it to be an effective candidate for microbe-assisted phytoremediation. This finding has great significance for the remediation of contaminated soil, especially soil with lead contamination, as *Sphingomonas* sp. ZYG-4 can assist the non-host plants in removing the pollutants, and it also has broad application prospects.

## 5. Conclusions

Endophytic bacteria isolated from *A. adenophora*, a pioneer plant in mining areas, exhibit the potential to enhance the growth of plants and facilitate heavy metal accumulation through the activation of various mechanisms crucial for promoting plant growth. Inoculation with *Sphingomonas* sp. ZYG-4, a selected strain with the best PGP potential, enhanced the phytoremediation potential of both host (*A. adenophora*) and non-host (*D. ambrosioides*) plants by promoting their Pb accumulation and biomass production. These results suggest that *Sphingomonas* sp. ZYG-4 shows potential as a valuable bioresource for improving phytoremediation in Pb contaminated soil. Further studies will investigate the effects of endophyte inoculation on the structure of rhizosphere bacterial communities and the underlying mechanisms in microbe-assisted plant heavy metal accumulation.

## Figures and Tables

**Table 1:** Plant growth promoting activities of the seven endophytic bacteria.

Isolates	Plant Growth Promoting Ability			
	IAA (mg·L^−1^)	ACC Deaminase Activity (µM α-KB·mg^−1^·h^−1^)	Phosphate Solubilization (mg·L^−1^)	Siderophore
XJR-7	35.7 ± 4.6 b	0.36 ± 0.07 ef	2.1 ± 0.4 d	−
XJR-9	18.6 ± 2.7 c	0.17 ± 0.04 f	9.6 ± 1.7 b	+
ZYG-1	19.1 ± 2.2 c	0.59 ± 0.13 e	20.8 ± 1.2 a	+
ZYG-4	53.2 ± 8.3 a	2.87 ± 0.19 a	11.2 ± 2.9 b	+
ZYG-6	3.1 ± 0.5 d	0.81 ± 0.11 d	1.9 ± 0.4 d	−
ZYG-7	2.5 ± 0.3 d	1.81 ± 0.21 b	2.5 ± 0.2 d	−
ZYG-10	20.8 ± 1.7 c	1.43 ± 0.09 c	5.7 ± 0.4 c	+

±, Standard deviation. The distinct letters denote significant differences at the 0.05 level, as determined by a one-way ANOVA followed by the Duncan’s test.

## Data Availability

The raw data supporting the conclusions of this article will be made available by the authors on request.

## Supplementary Materials

The following supporting information can be downloaded at: https://www.mdpi.com/article/10.3390/toxics12040291/s1, Table S1: Geochemical conditions of the Pb mine tailing; Methods S1: PCR amplification protocol; Methods S2: Characterization of PGP traits of *A. adenophora* endophytes.
