# Supplementary material for: Improving Lead Phytoremediation Using Endophytic Bacteria Isolated from the Pioneer Plant Ageratina adenophora (Spreng.) from a Mining Area
Source: Toxics. 2024 Apr 16;12(4):291. doi: 10.3390/toxics12040291 (PMC11054004; doi:10.3390/toxics12040291)
Supplement: Supplementary file 1 [file toxics-12-00291-s001.zip › toxics-2910388-supplementary.pdf]

Support information

Improvement of lead phytoremediation by endophytic bacteria isolated from the pioneer plant *Ageratina adenophora (Spreng.)* on mining area

Qiqian Li[1,2,4], Siyu Yao[3], Hua Wen[1], Wenqi Li[1], Ling Jin[3], Xiuxiang Huang[1,4*]

1. College of Chemical and Biological Engineering, Hechi University, Hechi 546300, China;
2. Guangxi Key Laboratory of Sericulture Ecology and Applied Intelligent Technology, School of Chemistry and Bioengineering, Hechi University, Hechi 546300, China;
3. Department of Civil and Environmental Engineering and Department of Health Technology and Informatics, The Hong Kong Polytechnic University, Hung Hom, Kowloon 999077, Hong Kong;
4. Guangxi Collaborative Innovation Center of Modern Sericulture and Silk, School of Chemistry and Bioengineering, Hechi University, Hechi 546300, China.)

* Correspondence: 14002@hcnu.edu.cn

OTULINE

1. **Table S1.** Geochemical conditions of the Pb mine tailing
2. **Methods S1.** PCR amplification protocol
3. **Methods S2.** Characterization of PGP traits of *A.adenophora* endophytes

Table S1 Geochemical conditions of the Pb mine tailing

Geochemical conditions	Concentration (mg/kg)
pH	7.5±0.4
TOC	75.1±8.8
TN	22.4±3.9
TP	1.05±0.26
Pb	1242±98
Cd	376±61
Cu	345±82
Zn	730±43
Sb	82±16
Ni	97±10
Sn	95±28
As	772±115

Data represent the mean± standard error (SE) of three replicates.

SM: Materials and Methods

Methods S1. PCR amplification protocol

The PCR system prepared in a total volume of 25.0 μL, comprising 12.5 μL of rTaq premix buffer, 0.5 μL of each primer, 1 μL of template DNA and 10.5 μL of ddH_2O. Amplification was performed using an ABI 2720 Thermal Cycler (Thermo Fisher Scientific, Inc.) with the following steps: 95°C for 5 min; 32 cycles at 95 °C for 1 min, 54 °C for 1 min, and 72 °C for 2 min; and a final extension at 72 °C for 10 min.

Methods S2. Characterization of PGP traits of *A.adenophora* endophytes

IAA

The IAA production of isolated *A.adenophora* endophytes was determined by colorimetric technique using Salkowski's reagent containing: 150 mL H_2SO_4; 250 mL of distilled water; 7.5 mL 0.5M $FeCl_3$. *A.adenophora* endophytes were cultured in sucrose minimal salts (SMS) medium supplemented with 500 mg·L^{-1} of tryptophan and then incubated at 30°C for a duration of 4 days at 180 rpm. Bacterial suspension was then centrifugated at 5,000 rpm and the 1 mL supernatant was mixed with 2 mL of Salkowski's reagent. IAA production was determined according to the optical density at 600 nm compared with a standard curve from a serial of known concentrations of IAA.

ACC deaminase activity

The ACC deaminase activity of cell-free extracts was determined by monitoring the amount of α-ketobutyrate generated by the enzymatic hydrolysis of ACC. To detect ACC deaminase activity, the endophytic strains were inoculated into tubes containing 10 mL of modified Dworkin and Foster (DF) medium(4.0 g KH_2PO_4, 6.0 g Na_2HPO_4, 0.2 g$MgSO_4 \cdot 7H_2O$, 2.0 g glucose, 2.0 g gluconic acid and 2.0 g citric acid with trace elements: 1 mg $FeSO_4 \cdot 7H_2O$, 10 mg H_3BO_3, 11.19 mg $MnSO_4 \cdot H_2O$, 124.6 mg $ZnSO_4 \cdot 7H_2O$, 78.22 mg $CuSO_4 \cdot 5H_2O$, 10 mg MoO_3, pH 7.2 and 3 mmol ACC as a nitrogen source.) Subsequent to incubation at 30°C for 72 hours, the bacterial cells were harvested via centrifugation at 7878 ×g for 10 minutes at 4°C. The pellets were resuspended in 600 ml of 0.1 MTris–HCl buffer (pH 8.5) and cells were disrupted by the addition of 30 ml of toluene and vigorous vortexing. After reaction of mixtures, containing 100 mL of cell suspension,10 mL of 0.5 mol ACC and 100 mL of 0.1 mol Tris–HCl buffer (pH 8.5), for 30 min at 30℃, 1 mL of 0.56 mol/L HCl was added, and the mixtures were centrifuged at 14,000 g for 5 min. The mixtures containing no cell suspension or no ACC were used as controls. Then, 150 mL of 0.1% 2,4-dinitrophenylhydrazine in 2 N HCl was added to 1 mL of the supernatant. The

mixtures were reacted for 30 min at 30℃, supplemented with 1 mL of 2 mol/L NaOH and assayed for α -ketobutyrate via determination of the optical density at 540 nm.

Phosphate solubilization

To estimate the phosphate solubilization, endophytes were spread onto Pikovskaya Medium (0.5 g $(NH_4)_2SO_4$, 0.1 g $MgSO_4$ • $7H_2O$, 0.02 g NaCl, 0.02 g KCl, 0.003 g $FeSO_4$ • 7H2O, 0.003 g $MnSO_4$ • H2O, 5 g Ca_3 $(PO4)_2$, 10.0 g glucose, 0.5 g yeast extract and 1000 mL distilled water) and cultured at 30°C for 3 days. Then, 5 mL aliquot was collected and the endophytes cells were removed by centrifugation at 10,000 g for 20 min. The soluble-free phosphate in culture supernatant was estimated from the absorbance values obtained using the calibration curve with KH_2PO_4 at 700 nm.

Siderophores

The secretion of siderophores by the endophytic strains was evaluated using the method developed by Schwyn and Neilands, which utilizes blue agar plates containing the dye chrome azurol S (CAS). CAS agar plate (60.5 mg CAS was dissolved in 50 ml water and mixed with 10 ml iron solution (1 mM FeCl3 • 6H20, 10 mM HCl). Under stirring this solution was slowly added to 72.9 mg HDTMA dissolved in 40 ml water. It was mixed with Nutrient agar (2% agar) in 1:3 proportion at the time of pouring) and picric acid dipped strip method, respectively. After spotting the endophytes on CAS agar plates, they were then incubated at 30 °C for a duration of 6 days. A positive indication of siderophore production was observed through the presence of orange halos surrounding colonies on the blue agar plates.
